# Modulation of STAT-1, STAT-3, and STAT-6 activities in THP-1 derived macrophages infected with two *Trypanosoma cruzi* strains

**DOI:** 10.3389/fimmu.2022.1038332

**Published:** 2022-10-26

**Authors:** Melissa Martins Oliveira, Camila Ramalho Bonturi, Bruno Ramos Salu, Maria Luiza Vilela Oliva, Renato Arruda Mortara, Cristina Mary Orikaza

**Affiliations:** ^1^ ¹Microbiology, Immunology and Parasitology Department, Escola Paulista de Medicina, Federal University of São Paulo - UNIFESP, São Paulo, Brazil; ^2^ ²Biochemistry Department, Escola Paulista de Medicina, Federal University of São Paulo - UNIFESP, São Paulo, Brazil

**Keywords:** STAT-1, STAT-3, STAT-6, *Trypanosoma cruzi*, THP-1, macrophages, co-infection

## Abstract

*Trypanosoma cruzi* is the causative protozoan of Chagas’ Disease, a neglected tropical disease that affects 6−7 million people worldwide. Interaction of the parasite with the host immune system is a key factor in disease progression and chronic symptoms. Although the human immune system is capable of controlling the disease, the parasite has numerous evasion mechanisms that aim to maintain intracellular persistence and survival. Due to the pronounced genetic variability of *T. cruzi*, co-infections or mixed infections with more than one parasite strain have been reported in the literature. The intermodulation in such cases is unclear. This study aimed to evaluate the co-infection of *T. cruzi* strains G and CL compared to their individual infections in human macrophages derived from THP-1 cells activated by classical or alternative pathways. Flow cytometry analysis demonstrated that trypomastigotes were more infective than extracellular amastigotes (EAs) and that strain G could infect more macrophages than strain CL. Classically activated macrophages showed lower number of infected cells and IL-4-stimulated cells displayed increased CL-infected macrophages. However, co-infection was a rare event. CL EAs decreased the production of reactive oxygen species (ROS), whereas G trypomastigotes displayed increased ROS detection in classically activated cells. Co-infection did not affect ROS production. Monoinfection by strain G or CL mainly induced an anti-inflammatory cytokine profile by decreasing inflammatory cytokines (IFN-γ, TNF-α, IL-1β) and/or increasing IL-4, IL-10, and TGF-β. Co-infection led to a predominant inflammatory milieu, with reduced IL-10 and TGF-β, and/or promotion of IFN-γ and IL-1β release. Infection by strain G reduced activation of intracellular signal transducer and activator of transcription (STAT) factors. In EAs, monoinfections impaired STAT-1 activity and promoted phosphorylation of STAT-3, both changes may prolong cell survival. Coinfected macrophages displayed pronounced activation of all STATs examined. These activations likely promoted parasite persistence and survival of infected cells. The collective results demonstrate that although macrophages respond to both strains, *T. cruzi* can modulate the intracellular environment, inducing different responses depending on the strain, parasite infective form, and co-infection or monoinfection. The modulation influences parasite persistence and survival of infected cells.

## Introduction

Chagas’ Disease (CD) is an anthropozoonosis caused by *Trypanosoma cruzi* protozoan. CD is endemic in 21 Latin American countries with an estimated 12,000−14,000 deaths annually. An estimated 6 million people are infected worldwide (1ߝ^4^–^6^).

*T. cruzi* is a digenetic parasite with a life cycle that comprises three distinct evolutionary forms: epimastigotes, metacyclic or blood trypomastigotes, and amastigotes (1, 7, 8). Trypomastigotes are the classic infective forms. However, extracellular differentiation of trypomastigotes or early cell rupture can release extracellular amastigotes (EAs), which can also infect host cells and sustain the parasite life cycle (revised in 9). *T. cruzi* is currently categorized into seven discrete typing units (4, 10). This pronounced genetic variability allows reinfections by different strains, also known as co-infections, polyparasitism, or mixed infections. Previous studies have described polyparasitism in insect vectors (11, 12), human patients (13–16), experimental infection and treatment effects in animal models (17–20). At endemic areas, the occurrence of co-infections is estimated between 9 and 57% (14-16, 21). This broad range of mixed infections reflects the great abundance of strains, besides, some factors may underestimate the polyparasitism numbers, like the difficulty of parasite detection in the host, especially at chronic phase of infection, and socioeconomic conditions intrinsically associated with neglected tropical diseases, such as CD. It is not clear whether the correlation of these strain groups and clinical symptoms are related (22) and whether they modulate disease progression in the host. If so, it would at least partially explain the wide range of outcomes in patients with CD.

Besides parasite intrinsic virulence genetic factors, mechanisms of the host cell response are also related to the control or aggravation of disease. Macrophages are the first defense cells to encounter the parasite (23, 24) and these cells are highly responsive to the surrounding milieu and develop specific and complex profiles, which are classically activated macrophages (M1) and alternatively activated macrophages (M2) (25–27; revised in 28). The latter can be subdivided into M2a, M2b, M2c, and M2d (27).

M1 macrophages have high phagocytic capacity and can be generated *in vitro* by monocytes stimulated by interferon-gamma (IFN-γ) and/or lipopolysaccharide (LPS) (29). M2 macrophages are commonly obtained by the polarization of monocytes with IL-4 and/or IL-13, which are linked to tissue repair and angiogenesis mechanisms (reviewed by 30). Generally, in the acute phase of infection, proinflammatory cytokines that include IFN-γ, tumor necrosis factor-alpha (TNF-α), IL-1β, and IL-12 are related to parasite control, and anti-inflammatory cytokines (IL-10, IL-4, and transforming growth factor-beta [TGF-β]) result in opposing actions (3, 31). During the chronic phase of infection, the imbalance of few anti-inflammatory stimuli with an exacerbated inflammatory response appears to lead to tissue lesions, as seen in chronic chagasic cardiomyopathy (32–34). Reactive oxygen species (ROS) and reactive nitrogen species, such as nitric oxide (NO), are important molecules implicated in killing several intracellular pathogens. This is despite the presence of *T. cruzi* antioxidant enzymes that can inactivate these molecules and favor parasite persistence inside the host cell (35, 36; revised in 37).

Cytokines can induce the Janus kinase/signal transducer and activator of transcription (JAK/STAT) pathway, in which STATs dimerize and translocate to the nucleus and promote or repress DNA transcription factors of genes related to immunological responses (38–40). STAT-1 can be phosphorylated in response to IFN-γ. Stahl et al. (41) and Kulkarni et al. (42) demonstrated the essential role of the IFN-γ/STAT-1 axis in the control of *T. cruzi* infection. Infection of cardiomyocytes with the Tulahuén strain of *T. cruzi* induced an anti-apoptotic effect in an IL-6/STAT-3-dependent manner (43). In contrast, de Souza etal. (44) reported that *T. cruzi* Dm28c provoked apoptosis in peritoneal macrophages, but the authors discussed that apoptosis may not be induced by *T. cruzi* depending on the parasite strain and host cell tissue. IL-6 is a cytokine associated with macrophage polarization to the M2 profile, specifically M2d (45), and downstream phosphorylation of STAT-3 that is linked to the promotion of cellular proliferation and reduction of apoptosis (46). IL-4 can cause STAT-6 activation that promotes an anti-inflammatory response that is important to counteract the excess of inflammatory stimuli but is also related to parasite persistence and spread (47, 48).

Considering the important role of macrophages in *T. cruzi* infection response and the paucity of studies involving co-infection with different strains, the present study evaluated the activation of STAT-1, -3, and -6 in polarized macrophages derived from THP-1 cells. The results of co-infection with G (TcI) and/or CL (TcVI) strains were compared to those with monoinfections by each strain.

## Methods

### Ethics statement

This study was approved by the Ethical Research Committee of Federal University of São Paulo - UNIFESP (number 8152030419) in April 2019.

### THP-1 cells, macrophages, and cytokine stimuli

THP-1 human monoblasts were kindly provided by Dr. Fatima Ribeiro-Dias, Universidade Federal de Goiás, Goiânia, Brazil. The cells were cultivated in RMPI 1640 (Vitrocell, Brazil) supplemented with 10 mM HEPES (Thermo Fisher Scientific, USA), 20 mM sodium bicarbonate, 2 mM L-glutamine, 25 mM glucose, 1 mM sodium pyruvate, 5 mM mercaptoethanol, 2 mM penicillin, 0.15 mM streptomycin (all from Sigma-Aldrich, USA), and 10% fetal calf serum (FCS, Vitrocell). Cells were maintained in T25 culture flasks (TPP, Swiss) at a cell density of 5 × 10^4^ to 1 × 10^6^ cells/mL. To obtain macrophages derived from THP-1 cells, the cells were plated (2 × 10^5^ cells/mL) in 6-, 12-, or 96-well plates (Corning, USA) and incubated for 48 h in complete medium supplemented with 100 ng/mL phorbol 12-myristate 13-acetate (PMA, Sigma-Aldrich) at 37°C in an atmosphere of 5% CO_2_. The wells were washed twice with Hanks Balanced Salt Solution (Sigma-Aldrich) supplemented with 2 mM sodium bicarbonate to remove PMA and unattached cells. The plates were further incubated for 48 h in complete medium at 37°C and 5% CO_2_. Macrophages were then activated with cytokines for 24 h as follows using: IFN-γ (20 ng/mL) + LPS (100 ng/mL), IL-4 (25 ng/mL), or IL-6 (50 ng/mL). One group was not exposed to cytokines (M0 phenotype, representing the basal control).

### Trypanosoma cruzi

*T. cruzi* G (49) and CL (50) strains were used in its wild type form (WT) for ROS assay or transfected with fluorescent plasmids harboring green fluorescent protein (GFP) (G-pTREX-GFP; 51) or DsRed (CL-pTREX-DsRed; Ferreira et al., 2016). Tissue culture-derived trypomastigotes (TCTs) of *T. cruzi* were obtained from the supernatant of Vero cells (Instituto Adolfo Lutz, São Paulo, Brazil). TCTs were cultured in RPMI 1640 supplemented with 2.5% FCS, 2 mM penicillin, 0.2 mM streptomycin, and 84 µM gentamicin (all from Sigma-Aldrich) at 37°C and 5% CO_2_. EAs were obtained as previously described (52–54). Briefly, TCTs were cultured in liver infusion tryptose medium (pH 5.8) for 14−16 h, followed by 1 h in RPMI 1640 supplemented with 10% FCS. Four groups were used for every experiment: non-infected cells (NI), cells infected with G-GFP (G), cells infected with CL-DsRed (CL), and cells coinfected with both *T. cruzi* strains (COI). WT strains were only used in the ROS assay, due to fluorescence interference on measurements, since the infectivity of transfected and WT parasites was similar, as previously described (51). Multiplicity of infection (MOI) for TCTs and EAs was 2:1 for G and 20:1 for CL. *T. cruzi* was incubated with the cells for 3 h, the wells were washed three to seven times to remove parasites that were not internalized and maintained for 48 h at 37 °C and 5% CO_2_ until analyses were performed.

### Analyses of infected cells and cell viabilities

Cells (2 × 10^5^) were seeded in 12-well plates and stimulated and/or infected as described above and detached using 0.2% trypsin and 0.02% EDTA. Aliquots were obtained to assess the cell viability of each group using Fixable Viability Dye eFluor 780 (eBiosciences, USA) according to the manufacturer’s instructions. The suspended cells were fixed for 15 min in 4% paraformaldehyde (PFA) in phosphate buffered saline (PBS), washed in MACS buffer (PBS containing 0.05% bovine serum albumin and 2 mM EDTA), and suspended in 300 µL of MACS buffer. Cell suspensions were analyzed by flow cytometry using a LSRFortessa flow cytometer (BD Biosciences, USA), acquiring 5000 events/sample, with the gating strategy illustrated in **Supplementary Figure 1S**. For compensation controls of fluorescence, monoinfected cells were used. Analyses were performed using BD FACSDiva 6.2 and FlowJo 10.8.1 software (BD Biosciences).

### Measurements of nitric oxide and cytokines

THP-1 cells were plated as described above 48 h post-infection (hpi). The medium was filtered through a 0.22 µm pore size filter (Millipore, USA) and frozen at –20°C until measurements were made. NO was analyzed using a model 280i NO analyzer (Zysense). For the measurement of cytokines, the samples were stored in 10 mM EDTA, 9 μM aprotinin, and 10 μM E-64 (all from Sigma-Aldrich) at –80°C. IFN-γ, TNF-α, IL-1β, IL-4, IL-6, IL-10, and IL-12p70 were analyzed using the MILLIPLEX^®^MAP Human Cytokine/Chemokin Magnetic Bead Panel (Merck Millipore). TGF-β was analyzed with the MILLIPLEX^®^MAP TGF-β1,2,3 Magnetic Bead Kit (Merck Millipore). All analyses used the MAGPIX^®^ system (Merck Millipore), according to the manufacturer’s instructions.

### Measurement of ROS

Cells (1 × 10^4^) were seeded in black 96-well plates and cultivated as previously described. We used the 2’,7’-dichlorofluorescein diacetate/2’,7’-dichlorodihydrofluorescein diacetate (DCFDA/H2DCFDA) − Cellular ROS Assay Kit (Abcam, UK) according to the manufacturer’s instructions. Measurements were performed at 3 and 24 hpi by fluorescence quantification of each cell using ImageJ v.1.53m software (NIH) from at least four images per group (40× magnification) using an Olympus IX70 inverted microscope. Data are presented as corrected total cell fluorescence (CTCF) calculated as integrated density – (area of selected cell × mean fluorescence of background readings). Analyses were performed using the ratio of cell CTCF and M0 mean CTCF.

### Evaluation of STATs

THP-1 cells (5.5 × 10^5^) were seeded into 6-well plates and stimulated as previously described. After 48 hpi, cells were detached with 300 µL of trypsin solution, inactivated with complete medium, and washed with MACS. Aliquots were reserved to test cell viability as described above. Cells were fixed with 4% PFA in PBS, incubated with 5% autologous serum, and permeabilized with PermBufferIII (BD Biosciences). The samples were incubated with combinations of two antibodies: anti-STAT-1 pY701 Pacific Blue and anti-STAT-1 pS727 AlexaFluor 647, or anti-STAT-3 pY705 Pacific Blue and anti-STAT-6 pY641 AlexaFluor 647 (all from BD Biosciences). As compensation controls, we used beads for STATs antibodies and monoinfected cells for G-GFP and CL-DsRed. A gating strategy (**Supplementary Figure 1S**) consisted of the selection of single cells, followed by macrophages, and then GFP × DsRed. Thus, each quadrant represents one type of infected cell (Q1 by G, Q2 coinfected, Q3 by CL, and Q4 NI cells). Within each quadrant, we analyzed the presence of phosphorylated STATs. Acquisition of 20.000 events/sample was performed with BD FACSDiva version 6.2 in a BD LSRFortessa flow cytometer. Median fluorescence intensity (MFI) of STATs were analyzed with FlowJo software version 10.8.1.

### Statistical analyses

All experiments were performed in duplicate or triplicate, with two or three biological replicates. Data are expressed as the mean ± standard deviation for all parameters except ROS; the data of the latter are expressed as standard error of the mean. Differences were considered significant at a p-value < 0.05. The determinations involved two-way ANOVA with Tukey’s post-test when samples presented a normal distribution and otherwise by Kruskal−Wallis with Dunn’s post-test calculated with GraphPad Prism 8.3.0. Data of the phosphorylated STATs are presented as heatmaps with the ratio of MFI in each condition in relation to M0 basal MFI.

## Results

### Macrophage infection is greater with *T. cruzi* G strain, but IL-4 favors infection by CL strain

**Figure 1:**
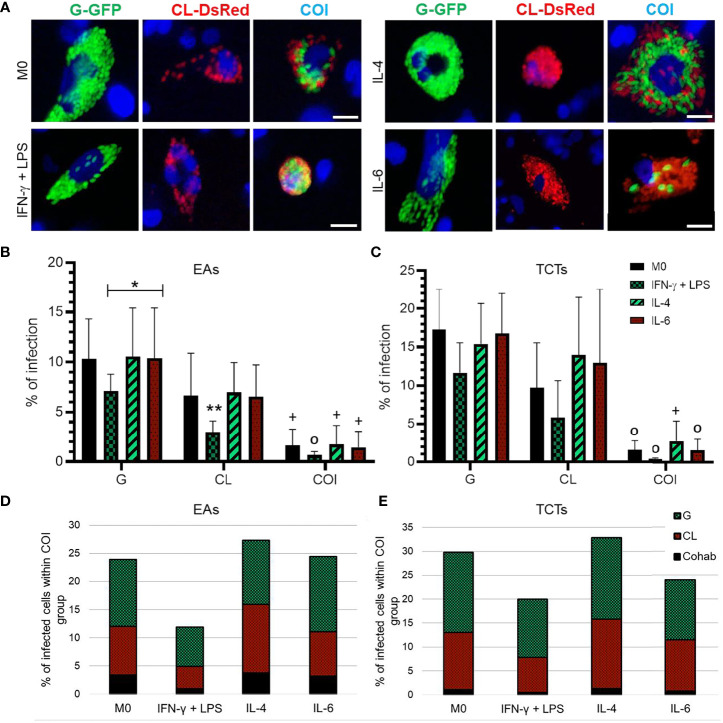
IFN-γ + LPS treatment reduced the number of infected macrophages by both strains, while IL-4 and IL-6 augmented CL infected cell numbers. **(A)** Representative images of *Trypanosoma cruzi* amastigotes in THP-1 derived macrophages at 72 h post-infection (hpi); Green = G-GFP; red = CL-DsRed; blue = cell nuclei stained with *Hoechst 33342*. COI = co-infection. Bar = 20 μm. Percentage of infected cells at 48 hpi analyzed by flow cytometry when cells were infected by extracellular amastigotes (EAs) **(B)**, or by tissue culture-derived trypomastigote forms (TCTs) **(C)**. Graphs represent mean and standard deviation of three independent experiments. *p < 0.05; **p < 0.01; +p < 0.05 COI *vs*. monoinfections of both strains; 0p ≤ 0.01 COI *vs*. G monoinfection; determined by two-way ANOVA with Tukey’s *post hoc*. Distribution of strains within co-infection group, cells only with the G strain were predominant, while CL strain was more present in the groups stimulated with IL-4 or IL-6 and cohabited cells (Cohab) presented lower numbers in the infection either by EAs **(D)** or TCTs **(E)**.

THP-1 derived macrophages were cultivated and stimulated with cytokines as described above. None of these treatments significantly interfered with cell viability in all groups, as evidenced by the cell viability rate >90% (**Supplementary Figure 2S** **)**. *T. cruzi* strains G and CL, and both TCTs and EAs were capable of infecting macrophages, independent of cytokine stimulus (**Figures 1A−C**). However, infection by TCTs presented higher infection rates at 48 hpi than by EAs. Macrophages stimulated with IFN-γ + LPS [M(IFN-γ)] led to lower numbers of infected cells in all conditions. Macrophages stimulated with IL-4 or IL-6 [M(IL-4) and M(IL-6), respectively] tended to augment CL-infected cells, mainly when infected with TCTs. Consequently, infected cells also increased in the co-infection group. This effect was more evident in M(IL-4). Among the infected cells in the co-infection condition, we could separate into G-only, CL-only, or cohabited cells, as shown in **Figures 1D, E**. We observed that only G cells were predominant in M0 and M(IFN-γ), and that CL macrophages appeared in almost the same proportion as G macrophages in M(IL-4). Despite this variance, cohabiting cells were rare under all conditions tested.

### NO levels vary in infection by CL, but not by G strain, otherwise G strain infection increased ROS in classically activated macrophages 

**Figure 2:**
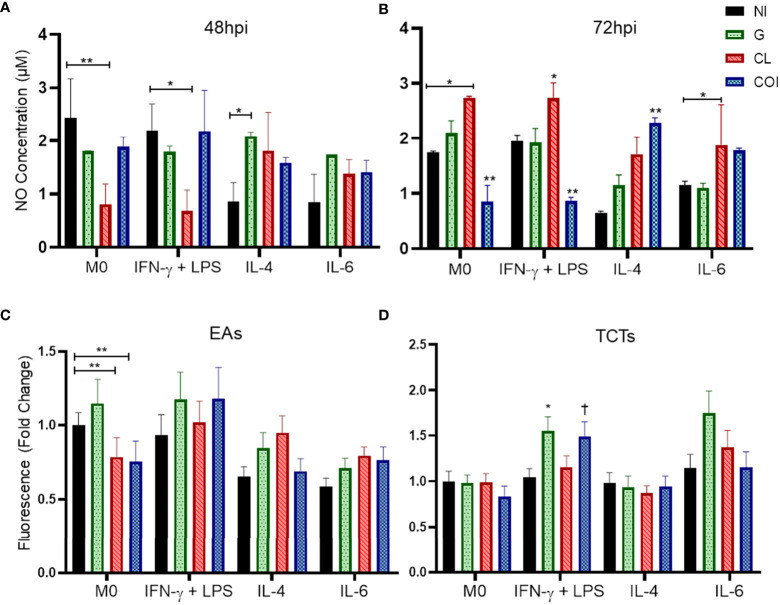
Nitric oxide (NO) concentration remains at stable levels when macrophages are infected by strain G, but not CL or co-infection (COI); otherwise, G infection promoted increase of reactive oxygen species (ROS) in cells stimulated with IFN-γ+LPS. CL monoinfection and co-infection showed an opposite pattern of NO production by non-polarized macrophages (M0) and cells stimulated with IFN-γ + LPS at the two evaluated times (48 and 72 hours post infection – hpi). Graphs represent the mean and standard deviation of duplicates from two independent experiments at 48 hpi **(A)** and 72 hpi **(B)**. Statistical differences determined by the two-way ANOVA test with Tukey’s *post hoc*, *p < 0.05; **p < 0.01. Extracellular amastigotes (EAs) of CL strain infection and co-infection in M0 produced less ROS than the uninfected macrophage **(C)**. Regarding TCTs, macrophages stimulated with IFN-γ + LPS displayed the highest production of ROS with G and COI **(D)**. Graphs summarize the observations of two experiments performed in duplicate, quantified at 3 hpi. Statistical differences determined by Kruskal−Wallis test with Dunn’s *post hoc*. *p < 0.05; **p < 0.01; †p < 0.05 *vs*. M0 COI.

According to the results presented above, TCTs had higher infection rates. Thus, we evaluated NO production by these cells at 48 and 72 hpi (**Figures 2A, B**). M0 and M(IFN-γ) groups showed no differences in NO detection patterns at 48 or 72 hpi. CL monoinfection diminished NO levels in M0 and M(IFN-γ) at 48 hpi, while at 72 hpi the levels were higher compared to their NI control, including M(IL-6). G monoinfection seemed to maintain stable levels of NO, even in M(IL-4) cells, which decreased NO in the NI group. After 72 hpi, COI decreased NO in M0 and M(IFN-γ) and increased in the M(IL-4) group.

ROS were evaluated at 3 and 24 hpi. No significant differences were observed at 24 hpi (**Supplementary Figure 3S**). At 3 hpi (**Figures 2C, D**), CL EAs alone or with strain G reduced ROS in M0. Otherwise, TCTs in M(IFN-γ) COI and G were higher than the same infection at M0 or M(IL-4), respectively.

### Monoinfections predominantly promoted decrease and co-infections increase of inflammatory cytokines release

**Figure 3:**
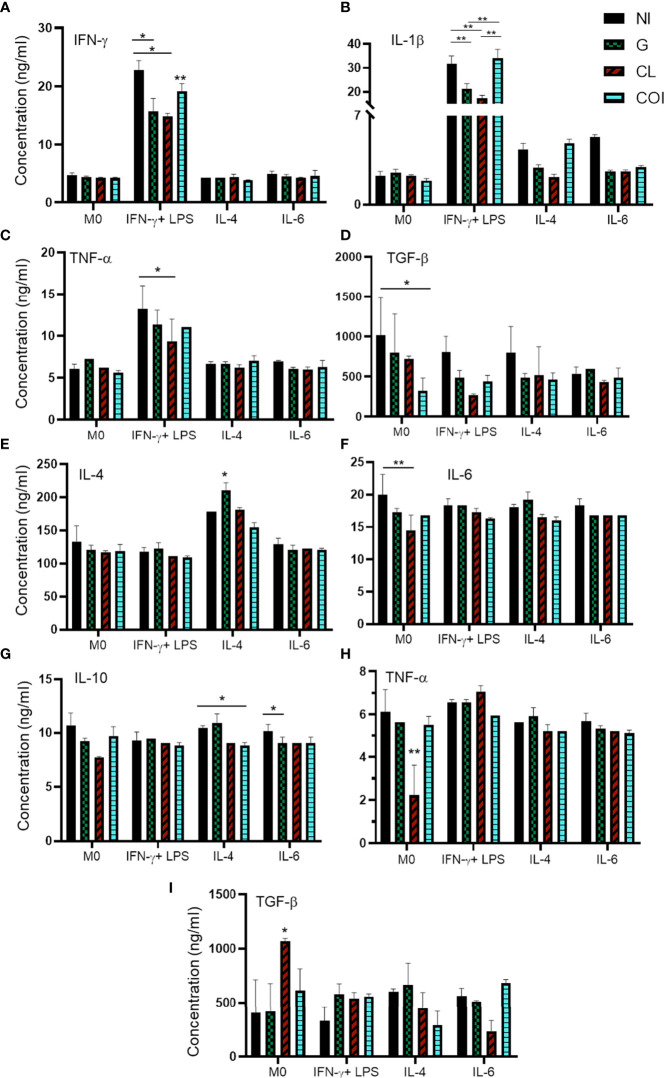
Quantification of cytokines in the supernatant of macrophages showing predominant decreased levels of proinflammatory cytokines in monoinfections and increased levels of inflammatory cytokines in co-infection. Macrophages were polarized and/or infected by extracellular amastigotes (EAs) **(A**–**D)** or by tissue-culture trypomastigotes (TCTs) **(E**–**I)**, and quantification was measured 48 h after infection. Graphs represent the mean and standard deviation of concentration values (ng/mL) from two measurements of each sample in duplicate, statistical differences determined by the two-way ANOVA test with Tukey’s *post hoc* test. *p < 0.05; **p < 0.01.

Another way to evaluate macrophage activation is through the secreted cytokines. Since M(IFN-γ) did not elevate NO levels as expected, we analyzed IFN-γ, TNF-α, IL-1β, IL-4, IL-6, IL-10, IL-12, and TGF-β. Unlike the NO measurements, we observed a higher release of proinflammatory cytokines (IFN-γ, IL-1β, and TNF-α) in the M(IFN-γ) group (**Figure 3**) and this was likely due to cell activation.

Monoinfection of EAs with G or CL reduced the levels of IFN-γ and IL-1β compared to those in NI cells among M(IFN-γ). Co-infection elevated the levels of both cytokines, especially IL-1β. Monoinfection with CL also diminished TNF-α levels in the same IFN-stimulated group. Co-infection significantly reduced TGF-β levels in the M0 group (**Figures 3A−D**). The other conditions did not produce significant differences in EAs infection (**Supplementary Figures 4S**).

Infection of TCTs led to different responses. CL monoinfection reduced TNF-α and IL-6 in addition to enhanced TGF-β levels in M0 cells. M(IL-4) resulted in increased IL-4 when infected with only the G strain and reduced IL-10 during co-infection. IL-10 was also reduced in the monoinfection of G when cells were stimulated with IL-6 (**Figures 3E−I**). The other conditions and cytokines did not show significant differences during infection of TCTs (**Supplementary Figure 5S**).

### Analysis of STATs activation

**Figure 4:**
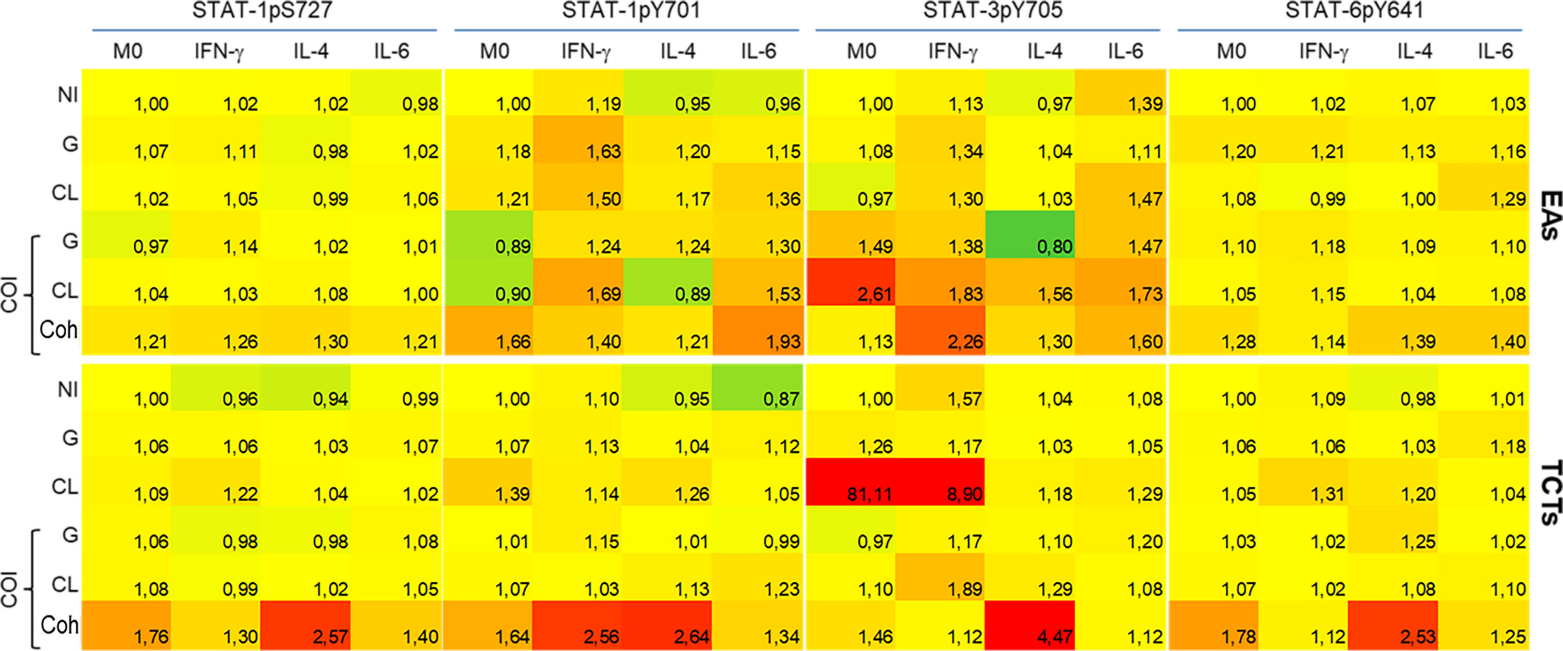
Coinfected cells induced increased phosphorylation of all STATs residues evaluated when compared to monoinfections at 48 h post infection. Activation of STATs were assessed by flow cytometry, with detection of phosphorylated Y701 (pY701) and pS727 of STAT-1, pY705 of STAT-3 and pY641 of STAT-6. Rows indicate infection groups: NI uninfected; G infection; CL infection; and subgroups within co-infection (COI) inside the brace. Coh: cohabited cells, presence of both strains in a cell. Top rows represent infection by extracellular amastigotes (EAs), and bottom rows by tissue-culture trypomastigotes (TCTs). Columns indicate the STATs residue evaluated and macrophage stimuli. The comparative activation is presented as green-red scale heatmap with the ratio of median fluorescence intensity (MFI) of each condition in relation to uninfected M0 basal MFI (ratio 1, yellow; <1 less phosphorylation = green; >1 more phosphorylation = red).

JAK-STAT is an important intracellular pathway activated by cytokines. Accordingly, we evaluated four domains of the three STATs at 48 hpi. The basal MFI of the M0 NI group was used as a reference (ratio 1, yellow) to evaluate the increase (>1, red) or decrease (<1, green) of the other MFIs (**Figure 4**). We observed that the activation effect due to the examined cytokine stimuli was still detectable, as observed on the NI line in M(IFN-γ) on STAT-1, M(IL-6) on STAT-3, and M(IL-4) on STAT-6. These results were obtained even though we could observe distinct activation patterns in each infection, depending on the infective form of the parasite.

The heatmap graphic enables two main comparisons: between lines (effect of infection within cytokine-treated group) or between columns (effect of cytokine stimuli within each infection). For our purposes, we made comparisons between lines, as our aim was to observe differences among mono- or co-infection. STAT-1pS727 and STAT-6pY641 were generally near basal levels, except for the EAs and TCTs infected with both parasites. Infected EAs displayed elevated activation of tyrosine residues of STAT-1 and STAT-3 in the presence of either parasite in M(IFN-γ) or M(IL-6) cells; the latter did not show this increase with G monoinfection. Infection of TCTs seemed to cause fewer alterations, but stronger effects on phosphorylation; CL monoinfection greatly increased STAT-3 activation, mainly in M0 and M(IFN-γ) groups. EAs and TCTs harboring both strains displayed the highest phosphorylation among the studied residues under almost all conditions.

**Figure 5:**
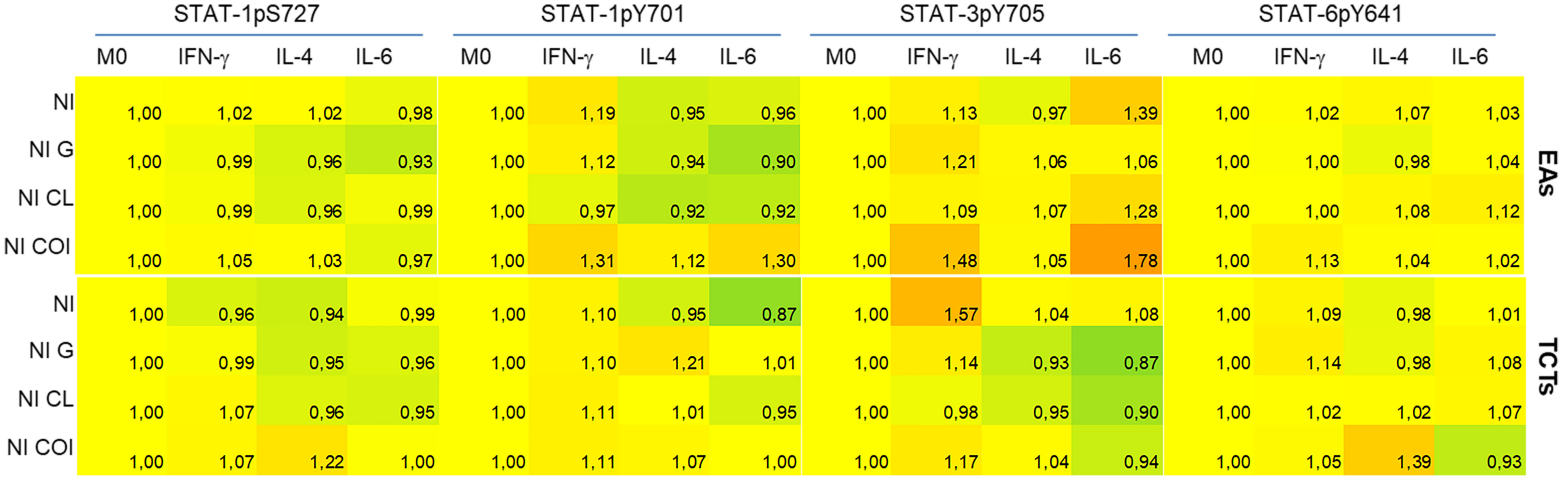
Uninfected cells (NI) demonstrated dephosphorylation of STATs, mainly in monoinfections, except for STAT-3 evaluated in cells infected by extracellular amastigotes (EAs). Activation of STATs were assessed by flow cytometry at 48 h post-infection, with detection of phosphorylated Y701 (pY701) and pS727 of STAT-1, pY705 of STAT-3 and pY641 of STAT-6 residues. Rows indicate the condition group: NI - uninfected; NI G - uninfected cells within G infected group; NI CL - uninfected cells within CL infected group; and NI COI uninfected cells within co-infected group. Top rows represent infection by extracellular amastigotes (EAs), and bottom rows by tissue-culture trypomastigotes (TCTs). Columns indicate the STATs residue evaluated and macrophage stimuli. The comparative activation is presented as green-red scale heatmaps with the ratio of each condition’s median fluorescence intensity (MFI) in relation to M0 basal MFI (ratio 1, yellow; <1 less phosphorylation = green; >1 more phosphorylation = red).

We also analyzed the uninfected cells within each infection condition, as illustrated by the Q4 quadrant in **Supplementary Figures 1S–C**. These results are presented in **Figure 5**, taking as reference the M0 NI condition and comparisons as mentioned in the previous figure. We observed minor effects that mostly tended to be dephosphorylation of STATs-1 in M(IL-4) or M(IL-6) cells. The exception was NI COI with EAs, which displayed higher activation of STAT-3 in M(IFN-γ) or M(IL-6) cells.

## Discussion

*T. cruzi* strains and vector diversity in endemic zones provide multiple contacts between humans and triatomines. The latter also have the chance to feed on the blood of numerous infected individuals (55, 56). This environment has a direct impact on evolution and parasite genetics. Reinfections in one individual can result in co-infection or mixed infection with more than one strain interacting in the same host. These events can influence symptoms, disease progression, and even treatment effectiveness (18).

Our observations demonstrate that both G and CL strains could infect macrophages, whether they were activated or not, independent of the infective form, either EAs or TCTs. The G strain is characterized by low infectivity of trypomastigotes while displaying highly infective EAs *in vitro*. CL metacyclic trypomastigotes present high infection rates and low infectivity as EAs in epithelial cell cultures (49; reviewed by 57). Interestingly, our results in a model of professional phagocytic cells showed that TCTs were more infective than EAs independent of strain, and strain G was able to infect a higher number of cells than strain CL. Even when using an MOI that was ten times higher, strain CL resulted in low numbers of infected THP-1-derived macrophages, except when cells were stimulated with IL-4 or IL-6, then, both strains presented similar infection rates (**Figure 1**). Owing to the multiclonal structure of *T. cruzi*, different isolates can exhibit symbiotic behavior. However, environmental stressors can also promote competition among them (55, 56), this fact may at least partially explain our low co-infection numbers.

**Figure 6:**
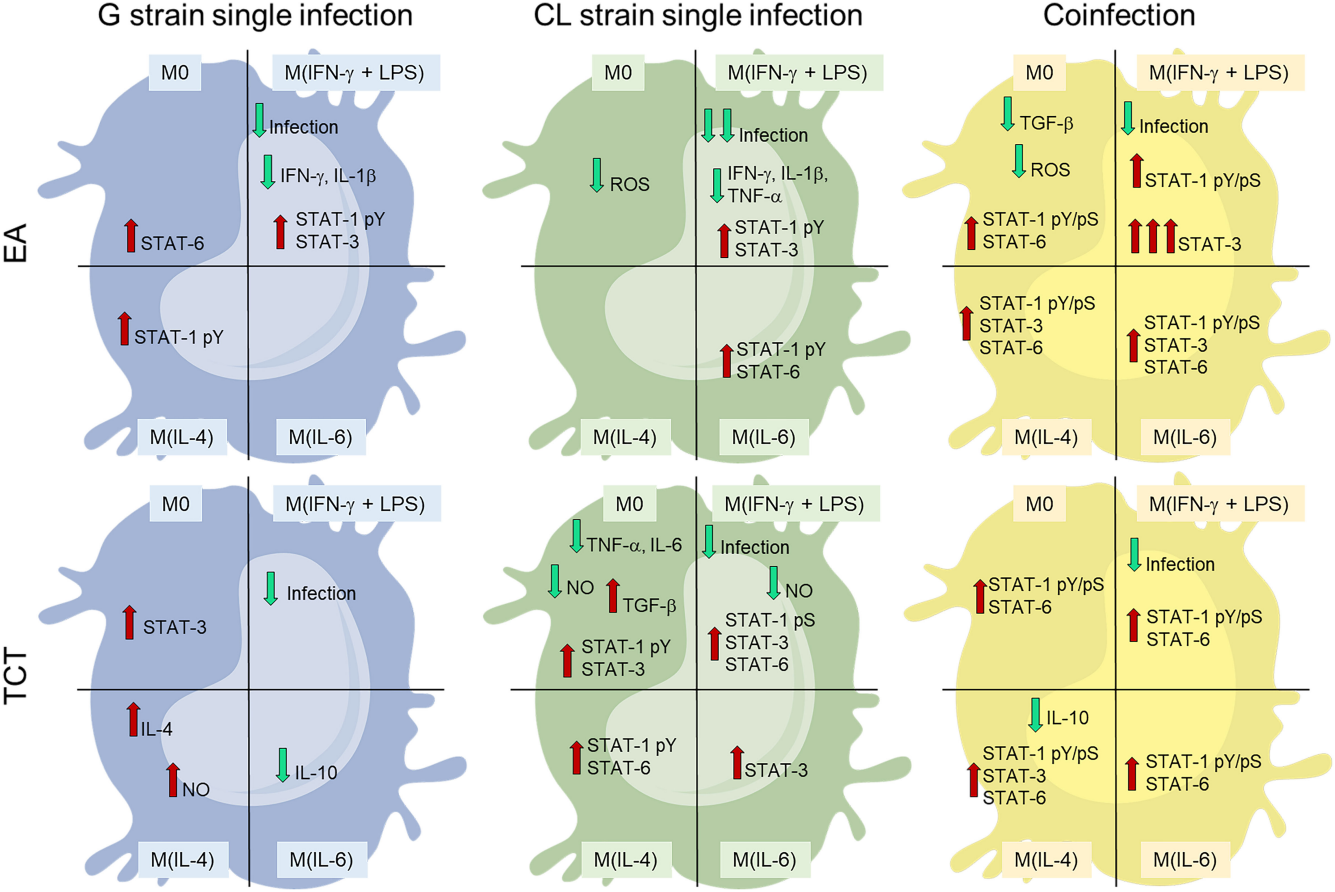
Monoinfection conditions of higher infected cell numbers [strain G or strain CL M(IL4)] demonstrated less host cell responses than co-infection (COI). COI promoted important activation of STATs and resulted in low infection rates. Cells are divided in four categories, representing the four stimuli states: M0 – without cytokine; M(IFN- γ+LPS) – stimulated with 20 ng/mL and 100 ng/mL, respectively; M(IL-4) – stimulated with 25 ng/mL of IL-4; or M(IL-6) – stimulated with 50 ng/mL of IL-6. The top cells represent infection by extracellular amastigotes (EAs). The bottom cells depict tissue cultured trypomastigotes (TCTs). Blue cells are infection by G strain, green cells by CL strain, and yellow cells by both strains. Green and red arrows denote decrease and increase, respectively.

The main differences between infected and NI cells at 48 hpi are shown in **Figure 6**. Infection with strain G caused less intracellular reaction than infection with CL. This may contribute to the thriving of G in this cell model. We observed that in the M(IL-4) condition, which favored CL infection, no or little intracellular disturbance was evident, this data corroborates that of Vaena de Avalos et al. (58). The latter authors concluded that *T. cruzi* infection in susceptible cells induced few transcriptional changes at early stages. Finally, we observed that co-infection resulted in a different pattern of responses from the cells with the activation of more STATs, independent of cytokine stimuli.

The number of infected cells decreased in M(IFN-γ) macrophages under all infection conditions, demonstrating the effectiveness of IFN-γ in hindering parasite entry. IFN-γ is a key cytokine in *T. cruzi* control. Its release is increased in the acute phase of infection, and this provides an inflammatory environment that elicits a Th1 response, these events are essential for the reduction of parasitemia and disease counteraction (24, 34, 59–62). An effector pathway that can be activated by IFN-γ is inducible NO synthase (iNOS). Subsequent NO production and other cytotoxic radicals are detrimental to *T. cruzi* and to the host (63, 64). However, some studies have also discussed the role of NO, which may need synergistic cytokine effects or cause tissue toxicity, or at low concentrations can promote growth and survival of intracellular pathogens (64–67). Nevertheless, we did not observe any increase in NO due to IFN-γ stimulation, even using a more sensible technique (chemiluminescent method) (**Figure 2**). However, recent studies argued that THP-1 cells are not good NO producers because macrophage differentiation with PMA treatment can exhaust NO production capacity (68–71). We observed that NO varied over time when infected by CL (low to high) or by both strains (high to low) with opposite patterns, whereas G maintained stable NO levels in M0 and M(IFN-γ) (**Figure 2**). It is possible that the intracellular amastigotes of CL interfere with the NO pathway, and CL growth in COI cells is delayed compared to that in infection alone. Zalloum etal. (72) demonstrated that *T. cruzi* could suppress NO release in BALB/c peritoneal macrophages. Depending on the strain, this effect was only seen in cells in contact with trypomastigotes on supernatants, so the release of trypomastigotes by an infected cell could cause downstream signaling to reduce NO production. de Castro Neto etal. (37) also reviewed several parasite antioxidant factors that can counteract the oxidative stress induced by *T. cruzi* infection. The expression of these proteins and enzymes can be specific to each parasite cycle stage, as it has to adapt its energetic requirements and immune response evasion.

Monoinfections seemed to induce an anti-inflammatory profile of cytokines, with decreased levels of IFN-γ, IL-6, IL-1β, and TNF-α, and increased levels of TGF-β and IL-4. These findings demonstrated that the establishment of infection, although with variations according to stimuli, induced a favorable environment for parasite persistence. Co-infection tended to promote inflammation with heightened IFN-γ and IL-1β and diminished IL-10 levels when compared to monoinfections.

Full activation of STAT-1 requires phosphorylation of both serine 727 and tyrosine 701 to enhance the transcription of innate immunity-related genes (41, 73). The only condition in which we detected both phosphorylated residues was on COI, independent of previous stimuli. Phosphorylation of Y701 residue was detected in almost all infected M(IFN-γ). This activation is essential for dimerization, stability, nuclear translocation, and enhanced transcriptional effects of STATs (74, 75). Phosphorylation of S727 is required to fully activate transcriptional effects and for pathogen control (73, 76, 77). STAT-1 has also been described as an important transcriptional factor for procaspases (76), thus, impairment of its full active state can inhibit intrinsic signaling for apoptosis.

STAT-3 can have opposite effects of STAT-1 on the cell cycle and apoptosis. The constitutive activation of STAT-3 has been described in various cancer cells, including downregulation of apoptotic gene transcription and upregulation of proto-oncogene expression (78–82). In this context, as STAT-1pS (phosphorylation of serine residue) is required to induce apoptosis in cardiomyocytes after stress, and intracellular *T. cruzi* amastigotes inhibited this domain activation (41, 83) plus the increased STAT-3 activation at M(IFN-γ) infected with EAs of G or CL strains, we suggest that these two distinct mechanisms act synergistically to increase the survival of infected cells. *T. cruzi* induces the activation of STAT-3 in different cell types in response to IL-6. However, TGF-β can also phosphorylate STAT-3 and cause positive feedback in the transcription of both TGF-β and itself (43, 84–88), consistent with our results in M0 infected with CL TCTs (**Figure 6**).

Stahl et al. (85) demonstrated that although Y strain-infected cardiomyocytes had higher pSTAT-3 levels, cells were more apoptotic compared to uninfected controls. The authors discussed that the interaction and equilibrium of STAT-1 and STAT-3 activation can determine cell survival or death in this model of *T. cruzi* infection. Indeed, activated STAT-3 can promote SOCS3 (suppressor of cytokine signaling 3) which can inhibit IFN-γ/STAT-1 signaling in *T. cruzi* infection (84), and STATs-1 and -3 can form heterodimers that can target different DNA biding sites than their homodimers (82). Only some co-infection conditions resulted in fully activated STAT-1 and phosphorylated STAT-3.

IL-4 is a critical cytokine that controls antibody production, inflammation, and allergy. IL-4 has a main role in Th2 reprogramming and alternatively activated macrophages, demonstrating some opposing actions compared to IFN-stimulated cells, which generate Th1 and classically activated macrophages (reviewed by 89). IL-4 promotes the resistance of macrophages to *T. cruzi* (Tulahuén strain), while *in vivo* reduced resistance to infection has been described by the same strain (90, 91). The findings highlight the complex interplay of cytokines and cells in parasite control.

STAT-6 activated by IL-4 and TGF-β induce T cells to produce IL-9 from naïve or Th2 cells (89). IL-9 has recently been described as an important cytokine that controls fibrosis in cardiac tissues of mice infected with *T. cruzi* (92). Tarleton et al. (47) demonstrated that STAT-6 knockout mice were more resistant to *T. cruzi* chronic infection. The mice displayed less inflammation in cardiac or muscle tissues and no detection of parasites *in situ*. These findings indicate the essential role of STAT-6 in stimulating the Th2 response, which contributes to parasite persistence and disease severity. STAT-6 phosphorylation was observed in THP-1 derived macrophages infected with *Toxoplasma gondii*, with an anti-apoptotic effect on the cells (93).

The altered activation state of STATs seen in uninfected cells could be caused by vesicles or proteins directly secreted from the parasite, or extracellular vesicles (EVs) generated by infected macrophages (94–97). Factors secreted by *T. cruzi* can modulate uninfected cell responses in a paracrine manner to parasite invasion. EVs also can have a proinflammatory effect *via* the binding of Toll-like receptor-2 (95, 98, 99). However, different strains have surface variations. Thus, EVs can be very heterogeneous with altered quantity or content, which can interfere with parasite virulence and infectivity (99, 100). Interestingly, Cronemberger-Andrade et al. (95) evaluated EVs from THP-1 derived macrophages infected with *T. cruzi* strain Y and observed a reduction in STAT-1 and STAT-3 transcripts. These findings are consistent with our findings of reduced activation of these proteins in neighboring uninfected cells (**Figure 5**).

The role of STATs has been studied in several conditions, including other neglected tropical diseases (67, 76, 93, 101–103). The present study demonstrates that the activation state of macrophages elicits different interactions and responses from the host cells to *T. cruzi.* Both infective forms induce phosphorylation of the STAT residues evaluated within 48 hpi to prolong the survival of infected cells. The present data increase the understanding of *T. cruzi* infection, especially polyparasitism. The latter has not been extensively studied and related data can help to understand the variability in prognoses, symptoms, and effectiveness of treatments available for patients with CD.

## Data availability statement

The original contributions presented in the study are included in the article/**Supplementary Material**. Further inquiries can be directed to the corresponding author.

## Author contributions

MeO, CO, and RM conceived the study and designed the experiments. MeO, CO, CB, BS, and MaO performed the experiments. MeO, CO, and RM interpreted results and wrote the manuscript. All the authors contributed to the study and approved the submitted version of the manuscript.

## Funding

This work was supported by Fundação de Amparo à Pesquisa do Estado de São Paulo (FAPESP, grants 2016/15000-4 and 2019/08933-2) Coordenação de Aperfeiçoamento Pessoal de Nível Superior (CAPES) and Conselho Nacional de Desenvolvimento Científico e Tecnológico (CNPq).

## Acknowledgments

We would like to thank Gabriela Dantas de Oliveira Vieira, who began this study, Dr. Milena Brunialti and Dr. Reinaldo Salomão for their kind support with flow cytometry equipment, and Wiley Editing Services, for the careful and thorough review of this manuscript.

## Conflict of interest

The authors declare that the research was conducted in the absence of any commercial or financial relationships that could be construed as a potential conflict of interest.

## Publisher’s note

All claims expressed in this article are solely those of the authors and do not necessarily represent those of their affiliated organizations, or those of the publisher, the editors and the reviewers. Any product that may be evaluated in this article, or claim that may be made by its manufacturer, is not guaranteed or endorsed by the publisher.
