# Supplementary material for: Modulation of STAT-1, STAT-3, and STAT-6 activities in THP-1 derived macrophages infected with two Trypanosoma cruzi strains
Source: Front Immunol. 2022 Oct 26;13:1038332. doi: 10.3389/fimmu.2022.1038332 (PMC9643828; doi:10.3389/fimmu.2022.1038332)
Supplement: Supplementary file 1 [file DataSheet_1.pdf]

Supplementary Data

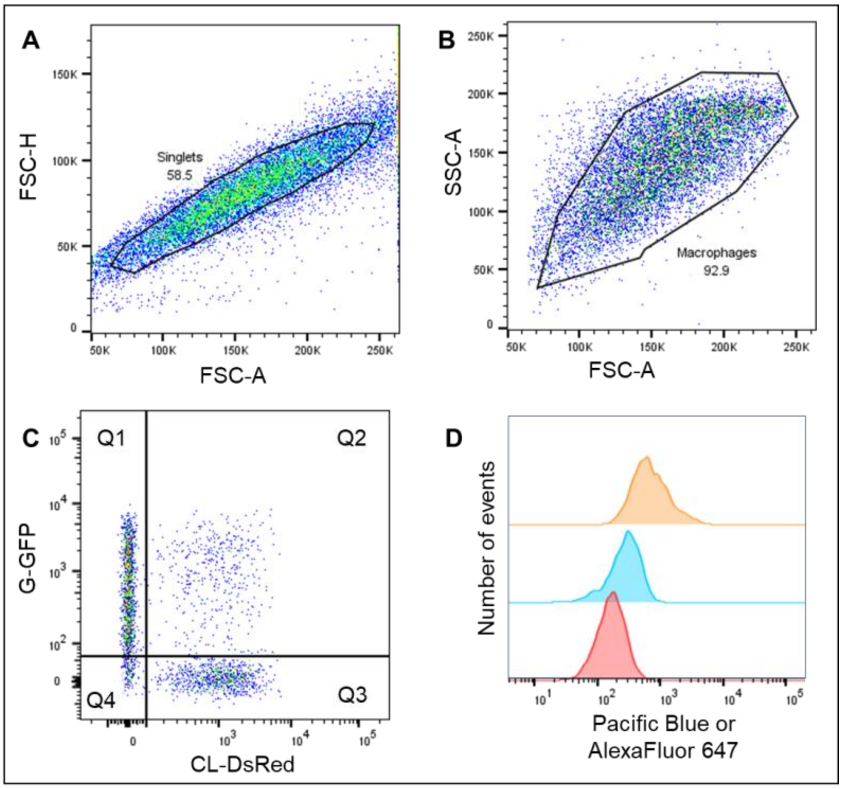

Supplementary Figure 1S – Gating strategy for flow cytometry analysis. A) Selection of single cells or singlets; B) Gate of macrophages; C) Identification of G-GFP infected cells (Q1), CL-DsRed infected cells (Q3), cells infected by both strains (Q2) and uninfected cells (Q4). Each of the previous quadrants are then evaluated for Pacific Blue or AlexaFluor 647; antibodies used were anti-STAT-1pY701 Pacific Blue with anti-STAT-1pS727 AlexaFluor 647 and anti-STAT-3pY705 Pacific Blue with anti-STAT-6pY641 AlexaFluor647. D) The histograms show different medians of fluorescence intensity, the upper denotes higher signal than the lower.

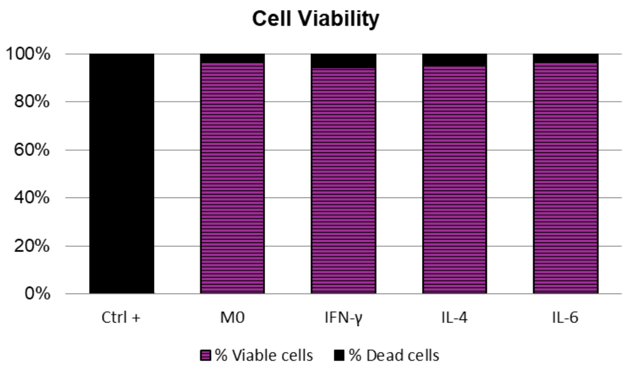

Supplementary Figure 2S – Viabilities of THP-1 derived macrophages were all above 90%, independently of cytokine stimuli. Cell viabilities were assessed by flow cytometry with Fixable Viability Dye eFluor 780 (eBiosciences). Ctrl + was paraformaldehyde-fixed cells stained as positive control for the dye; M0 = basal control cells; IFN-γ = IFN-γ 20 ng/mL + LPS 100 ng/mL; IL-4 (25 ng/mL); and IL-6 (50 ng/mL).

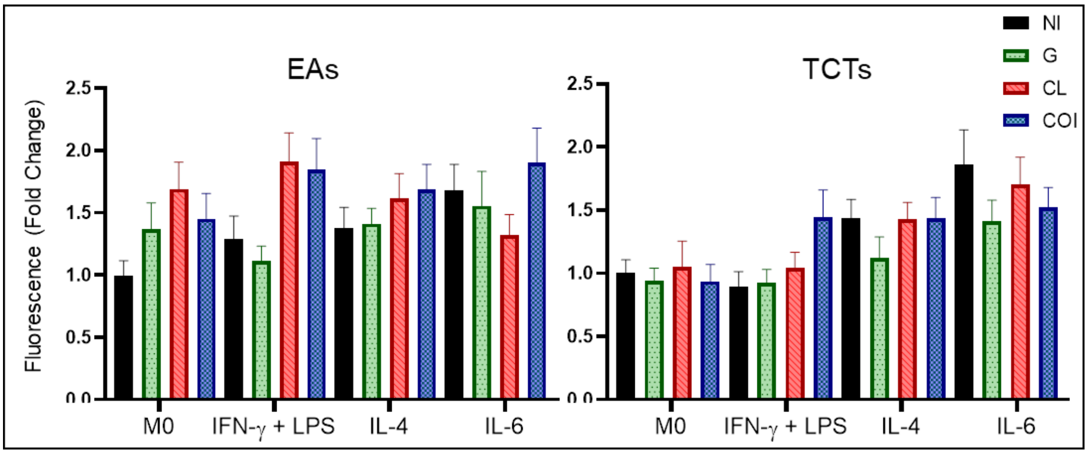

Supplementary Figure 3S – No significant difference in the detection of reactive oxygen species (ROS) production at 24 hours post infection. THP-1-derived macrophages were seeded in black-96-well plates. Macrophages were maintained in culture media M0 = basal control cells; or stimulated with IFN-γ = IFN-γ 20 ng/mL + LPS 100 ng/mL; IL-4 (25 ng/mL); or IL-6 (50 ng/mL). ROS detection was performed with2',7'-dichlorofluorescein diacetate/2',7'-dichlorodihydrofluorescein diacetate (DCFDA/H2DCFDA) – Cellular ROS Assay Kit (Abcam). Fluorescence quantification was acquired using ImageJ v.1.53m software (NIH) from at least four images per group (40× magnification) using an Olympus IX70 inverted microscope. Data are presented as corrected total cell fluorescence (CTCF) calculated as integrated density – (area of selected cell × mean fluorescence of background readings). Analyses were performed using the ratio of cell CTCF and M0 mean CTCF.

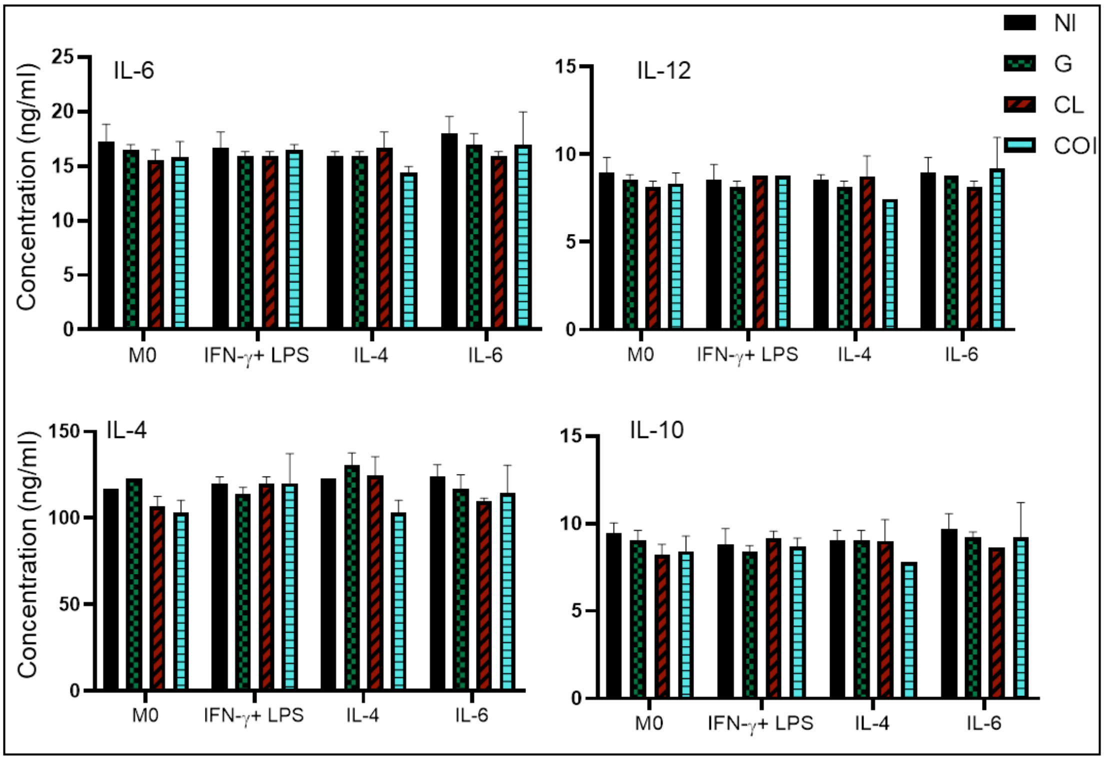

Supplementary Figure 4S – No significant difference in these cytokines levels in the supernatant of THP-1-derived macrophages. Macrophages were maintained in culture media M0 = basal control cells; or stimulated with IFN-γ (20 ng/mL) + LPS (100 ng/mL); IL-4 (25 ng/mL); or IL-6 (50 ng/mL). Cells were infected by extracellular amastigotes of strain G, CL or both (COI) and quantification was measured 48 h after infection by magnetic beads panels

MilliplexMap (Merck Millipore). NI = uninfected cells. Graphs represent the mean and standard deviation of concentration values (ng/mL) from two measurements of each sample in duplicate.

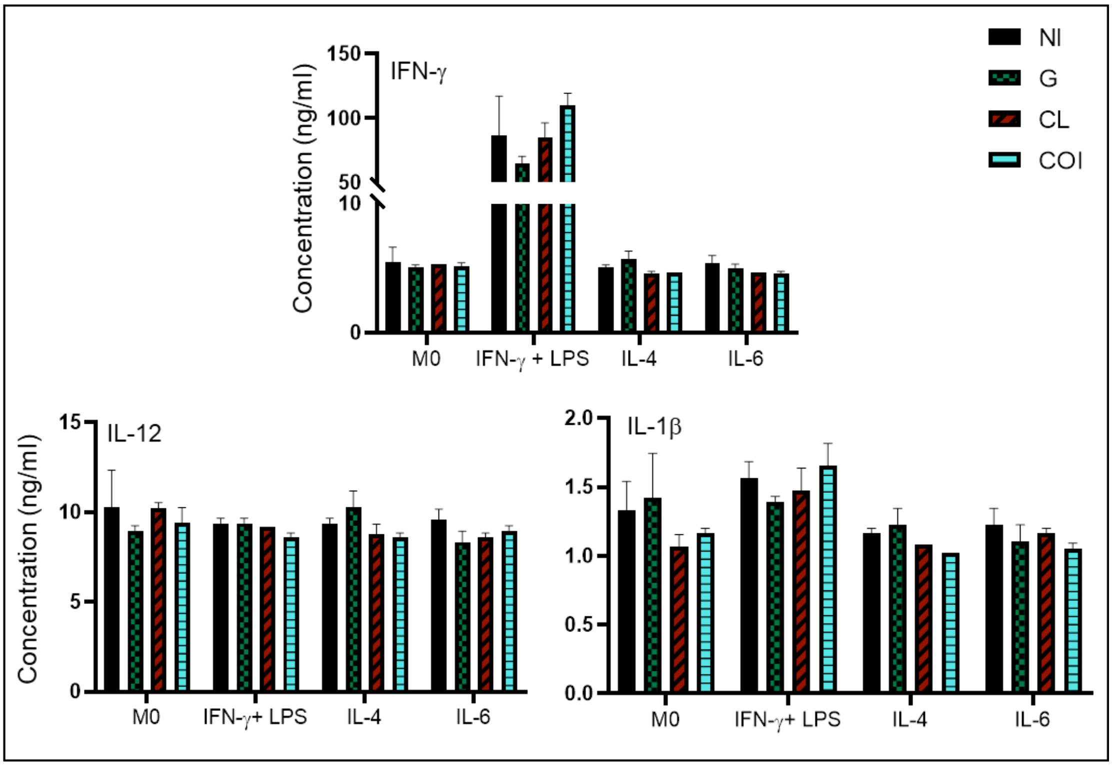

Supplementary Figure 5S – No significant difference in these cytokines levels in the supernatant of THP-1-derived macrophages. Macrophages were maintained in culture media M0 = basal control cells; or stimulated with IFN-γ (20 ng/mL) + LPS (100 ng/mL); IL-4 (25 ng/mL); or IL-6 (50 ng/mL). Cells were infected by tissue cultured trypomastigotes of strain G, CL or both (COI) and quantification was measured 48 h after infection by magnetic beads panels MilliplexMap (Merck Millipore). NI = uninfected cells. Graphs represent the mean and standard deviation of concentration values (ng/mL) from two measurements of each sample in duplicate.

Datasets can be found on online repository of Federal University of São Paulo at: https://repositorio.unifesp.br/bitstream/handle/11600/63177/TCC_Biomed_240222.pdf?sequence=1&isAllowed=y
